# Development of an antimicrobial resistance plasmid transfer gene database for enteric bacteria

**DOI:** 10.3389/fbinf.2023.1279359

**Published:** 2023-11-14

**Authors:** Suad Algarni, Steven L. Foley, Hailin Tang, Shaohua Zhao, Dereje D. Gudeta, Bijay K. Khajanchi, Steven C. Ricke, Jing Han

**Affiliations:** ^1^ Division of Microbiology, National Center for Toxicological Research, Food and Drug Administration, Jefferson, AR, United States; ^2^ Cellular and Molecular Biology Graduate Program, University of Arkansas, Fayetteville, AR, United States; ^3^ Division of Bioinformatics and Biostatistics, National Center for Toxicological Research, Food and Drug Administration, Jefferson, AR, United States; ^4^ Office of Applied Science, Center for Veterinary Medicine, Food and Drug Administration, Laurel, MD, United States; ^5^ Meat Science and Animal Biologics Discovery Program, Animal and Dairy Sciences Department, University of Wisconsin, Madison, WI, United States

**Keywords:** plasmids, transfer genes, database, conjugation, WGS analyses tools, Enterobacteriaceae

## Abstract

**Introduction:** Type IV secretion systems (T4SSs) are integral parts of the conjugation process in enteric bacteria. These secretion systems are encoded within the transfer (*tra*) regions of plasmids, including those that harbor antimicrobial resistance (AMR) genes. The conjugal transfer of resistance plasmids can lead to the dissemination of AMR among bacterial populations.

**Methods:** To facilitate the analyses of the conjugation-associated genes, transfer related genes associated with key groups of AMR plasmids were identified, extracted from GenBank and used to generate a plasmid transfer gene dataset that is part of the Virulence and Plasmid Transfer Factor Database at FDA, serving as the foundation for computational tools for the comparison of the conjugal transfer genes. To assess the genetic feature of the transfer gene database, genes/proteins of the same name (e.g., *traI/*TraI) or predicted function (VirD4 ATPase homologs) were compared across the different plasmid types to assess sequence diversity. Two analyses tools, the Plasmid Transfer Factor Profile Assessment and Plasmid Transfer Factor Comparison tools, were developed to evaluate the transfer genes located on plasmids and to facilitate the comparison of plasmids from multiple sequence files. To assess the database and associated tools, plasmid, and whole genome sequencing (WGS) data were extracted from GenBank and previous WGS experiments in our lab and assessed using the analysis tools.

**Results:** Overall, the plasmid transfer database and associated tools proved to be very useful for evaluating the different plasmid types, their association with T4SSs, and increased our understanding how conjugative plasmids contribute to the dissemination of AMR genes.

## Introduction

With the increasing utilization of DNA sequencing for the characterization of bacterial pathogens, such as *Salmonella* and other enteric pathogens, improved analysis tools are needed to more fully capture the value of the data which can contribute to improved public health. Whole genome sequencing (WGS) is being widely used by academic, public health and regulatory laboratories to better understand microbial genetics, physiology and evolution. *Salmonella enterica*-related infections alone affect millions of humans and animals in both developed and under-developed countries, which costs the global economy billions of dollars; therefore, tools for molecular-epidemiological studies that can improve prevention and treatment approaches are critically needed (Zafar et al., 2019). *Salmonella* strains can be typed into more than 2,600 serotypes based on their surface antigen profiles. However, the majority of human infections in the United States (U.S.) are associated with a smaller number of serotypes and the top five serotypes are including Typhimurium, Enteritidis, Newport, Javiana and Heidelberg (Algarni et al., 2022b).

Among *Salmonella* and related enteric pathogens, antimicrobial resistance (AMR) is a significant concern due to potential treatment failures. Often, AMR genes are encoded on transmissible plasmids; as such, research on plasmids has mainly been focused on a limited number of conjugative plasmids types that are associated with the multidrug resistance (MDR) in bacterial pathogens (Algarni et al., 2022b). Conjugative plasmids are known to be self-transmissible and encode genes associated with mobilization (MOB) and/or DNA transfer and replication (Dtr). The Dtr factors are important for the formation of the mating-pair apparatus and initiating DNA transfer that originates from the relaxases at the origin of transfer (OriT). The structural machinery associated with conjugal transfer in Gram-negative organisms is a type IV secretion system (T4SS) that is encoded by multiple genes carried on the plasmid. T4SSs are known to be central to the spread of several genetic determinants among bacterial strains. T4SSs contain 11 to 13 core proteins that form structural and energetic elements that facilitate the transmission of DNA or proteins directly from the cytoplasm of one bacterium to that of the recipient cell, which is in conjugation with another bacterium (Lawley et al., 2003). Plasmid mobility can be essential for the survival of microorganisms, as they can acquire factors to respond to a number of external stresses and understanding the distribution and epidemiology of plasmids is very important (Tamminen et al., 2012).

As WGS has been widely adapted as a basic laboratory technique in many public health laboratories, DNA sequences have become widely available for many bacterial isolates. These extensive sequencing efforts, coupled together with the development of bioinformatics tools, allow for an improved ability to discern bacterial strain transmission dynamics and resistance plasmid spread across strains, which enables investigators to increase their understanding of gene mobility among plasmids (Tamminen et al., 2012; Orlek et al., 2017). In addition to their role in plasmid mobilization, MOB genes can be used for multiple functions including the identification, classification and typing of plasmids based on the mobilization of relaxase genes (Algarni et al., 2022b; Chen et al., 2022). Besides MOB typing, additional plasmid classification schemes, such as replicon and plasmid taxonomic unit (PTU) typing schemes are useful for providing insights into the epidemiology of plasmid-mediated AMR (Carattoli et al., 2014; Orlek et al., 2017; Redondo-Salvo et al., 2021).

While these typing approaches provide data on the plasmid classification, an area where increased value can be obtained is by better understanding the diversity and functionality of the genes that make up the functional T4SSs which facilitate the transfer of plasmids from a donor to recipient bacterium. To enable this analysis of the transfer-associated genes, the current project was undertaken to identify and catalog the transfer-associated genes that are part of specific conjugal T4SSs in key groups of AMR plasmids. A resistance plasmid transfer gene database was developed along with computational tools that can be utilized for transfer gene identification in the *Enterobacteriaceae* and allow for a comparison of the different plasmid gene profiles among different isolates. This database was used to characterize strains and identify potential utility for predicting the factors that contribute to the spread of virulence and AMR-associated plasmids. Several key attributes are important to utilize the database for the characterization of plasmid genes. These attributes include that the sequences in the database that can distinguish among the different plasmid replicon types, that the transfer genes detected correspond to the predicted replicon types, and that the data can be utilized to better understand the genetics of the plasmids present in the strains. To these ends, several analyses were undertaken to assess the functionality and utility of the database including a comparison of the relatedness of genes with similar names or predicted functions (e.g., VirD4-like ATPases of T4SSs) utilizing multiple alignment of nucleic acid sequences or/and protein primary sequences (Chang and Wang, 2011; Orlek et al., 2017). In addition to the database, tools were developed to identify the specific plasmid transfer genes present in individual strains and to facilitate comparison of the genes between multiple strains carrying plasmids. The plasmid transfer factor database, along with transfer assessment bioinformatics tools, will also help to develop a strategy to prevent and combat highly mobilized MDR plasmids from further spreading in the environment.

## Methods and materials

### Generation of non-redundant, comprehensive list of plasmid-transfer associated genes from enteric bacteria

Based on the literature of the different plasmid incompatibility (Inc) groups that are most commonly associated with carrying and transmitting AMR genes in *Salmonella enterica* and related members of the *Enterobacteriaceae* (Carattoli, 2009; Rozwandowicz et al., 2018), the following Inc groups were selected for inclusion in the initial database: IncA/C, B/O, FIA, FIB, HI1, HI2, I1, I2, K, M, N, P, and W (note B/O and K were later combined). To generate a non-redundant, comprehensive list of plasmid transfer factors, a multistep process was utilized. In the first step, the GenBank nucleotide database (https://www.ncbi.nlm.nih.gov/nuccore/) was searched using the terms “Enterobacteriaceae and the specific plasmid type (e.g., “IncA/C″) (accessed in April, 2020). From the list of plasmids returned, those sequences that were in the range of size of the predicted plasmids (∼50–400 kb) and listed as “circular” were selected for further analyses. DNA sequences from over 1,200 plasmids representing the included plasmid types were downloaded as GenBank (.gb) formatted files and the files were processed for further analysis using the Feature Extract 1.2 server (https://services.healthtech.dtu.dk/service.PhP?FeatureExtract.1.2) to parse out the sequences of the different coding genes in the selected plasmid types (Wernersson, 2005). The parsed data was transferred to Excel for sorting and further assessment. Based on the annotations of the gene products, the transfer-associated genes for representative plasmids were identified and compared to other plasmids of the same Inc type to determine a plasmid-type specific, non-redundant list of putative transfer-related genes (Table 1; Supplementary Table S1). Representative plasmids identified for each of the different Inc types carrying the cohort of sequences were used to select the reference sequences for inclusion in the plasmid transfer gene dataset of the FDA Virulence and Plasmid Transfer Factor Database. Source isolates of the selected plasmids used in the generation of the Plasmid Transfer Factor Database are listed in Table 2.

**TABLE 1:** Transfer genes detected in the representive plasmid types.

Transfer Genes	IncA/C	IncB/O/K	IncFIA	IncFIB	IncHI1	IncHI2	IncI1	IncI2	IncM	IncN	IncP	IncW
*eex*										X		
*htdA*					X	X						
*htdF*					X	X						
*htdK*					X	X						
*htdO*					X	X						
*htdT*					X	X						
*htdV*					X	X						
*kikA*										X		
*korA*										X		
*korB*										X		
*mobA*									X			
*mobB*									X			
*mobC*									X			
*orf4*						X						
*orf9*						X						
*orf16*						X						
*orf17*						X						
*pilI*		X					X					
*pilJ*							X					
*pilK*		X					X					
*pilL*		X					X	X				
*pilM*		X					X	X				
*pilN*		X					X	X				
*pilO*		X					X	X				
*pilP*		X					X	X				
*pilQ*		X					X	X				
*pilR*		X					X	X				
*pilS*		X					X	X				
*pilT*		X					X	X				
*pilU*		X					X	X				
*pilV*		X					X	X				
*traA*	X		X	X			X			X	X	
*traB*	X	X	X	X	X		X	X		X	X	
*traC*	X	X	X	X	X		X	X		X	X	
*traD*	X		X	X	X		X	X		X	X	
*traE*	X	X	X	X			X	X		X	X	
*traF*	X	X	X	X	X	X	X			X	X	
*traG*	X		X	X	X	X	X	X		X	X	
*traH*	X	X	X	X	X	X	X	X	X		X	
*traI*	X	X	X	X	X	X	X	X	X	X	X	
*traJ*	X	X		X	X	X	X	X	X	X	X	
*traK*	X	X	X	X			X	X	X	X	X	
*traL*	X	X	X	X			X	X	X	X	X	
*traM*	X	X		X			X		X	X	X	
*traN*	X	X	X	X			X		X		X	
*traO*	X	X					X		X	X	X	
*traP*	X	X	X	X			X		X			
*traQ*	X	X	X	X			X		X			
*traR*	X	X	X	X			X		X			
*traS*	X	X		X			X					
*traT*	X	X	X	X			X					
*traU*	X	X	X	X			X		X			
*traV*	X	X	X	X			X					
*traW*	X	X	X	X			X		X			
*traX*		X	X	X			X		X		X	
*traY*		X		X			X		X			
*trbA*		X	X	X			X		X		X	
*trbB*		X	X	X			X		X		X	
*trbC*		X	X	X			X		X		X	
*trbD*			X	X							X	
*trbE*			X	X							X	
*trbF*			X	X							X	
*trbG*				X							X	
*trbH*											X	
*trbI*			X	X							X	
*trbJ*			X					X			X	
*trbL*								X			X	
*trbM*											X	
*trbN*					X						X	
*trhA*					X	X						
*trhB*					X	X						
*trhC*					X	X						
*trhE*					X	X						
*trhF*	X				X	X						
*trhG*						X						
*trhH*						X						
*trhI*					X	X						
*trhK*					X	X						
*trhL*					X	X						
*trhN*					X	X						
*trhO*					X	X						
*trhP*						X						
*trhR*					X	X						
*trhU*					X	X						
*trhV*					X	X						
*trhW*					X	X						
*trhY*					X	X						
*trhZ*						X						
*trwA*												X
*trwB*												X
*trwC*												X
*trwD*												X
*trwE*												X
*trwF*												X
*trwG*												X
*trwH*												X
*trwI*												X
*trwJ*												X
*trwK*												X
*trwL*												X
*trwM*												X
*trwN*												X

**TABLE 2:** Source isolates used in the generation of the plasmid transfer factor database.

Plasmid Type	Accession #	Host Species	Plasmid Name
IncA/C	NC_012692	*Escherichia coli*	pAR060302
	AP014634	*Salmonella enterica*	pSO4-21-304
	NZ_MN175387	*Klebsiella pneumoniae*	KP-14-6
IncB/O/K	MK088173	*Salmonella enterica*	R805a
IncFIA	NZ_CP014498	*Escherichia coli*	pZH193
IncFIB	J01724	*Escherichia coli*	plasmid F
IncHI1	KP899804	*Salmonella enterica*	pF8475
	KY320277	*Leclercia adecarboxylata*	pLec-476
IncHI2	MH287084	*Escherichia coli*	pSDE-SvHI2
IncI1	AP005147	*Salmonella enterica*	R64
IncI2	LN623683	*Salmonella enterica*	STH21_InHI2
IncK	NC_014477	*Escherichia coli*	pCT
IncM	KM406490	*Salmonella enterica*	pSEM
	CP029447.1	*Serratia marcescens*	pCAV1761-73
IncN	JX065631	*Escherichia coli*	pKT58A
IncP	KX377410	*Klebsiella pneumoniae*	pMCR_1511
	L10329.1	*Escherichia coli*	RP4
IncW	BR000038	*Escherichia coli*	R388
	EF633507	*Salmonella enterica*	pIE321

### Development of the comprehensive plasmid transfer gene dataset within the FDA virulence and plasmid transfer factor database

The gene-level nucleotide and amino acid sequence data and other related information (e.g., source, position, locus tag, product, DB_Xref, location and any notes) for each transfer factor was extracted from GenBank using a customized Python program. The extracted data was subsequently checked manually by our experts for accuracy, normalized to a delimited form and imported into the PostgreSQL relational database. The curated data were further processed by another customized Python program and local blast database was then created with makeblastdb tool from NCBI. A user-friendly website that hosts the data and tools was built using Django v2.2 and running on Apache HTTP Server v2.4.37. The database and tools described below are available at https://virulence.preprod.fda.gov/ (author’s note: this is the address for the preproduction site for evaluation of the database, upon completion of the peer review the site will be moved into production at the address - https://virulence.fda.gov/).

### Phylogenetic analyses of the transfer genes

The nucleotide sequences for each of the identified transfer factors (Supplementary Table S1) were imported into Microsoft Word and reformatted into the FASTA format, with each gene as an individual entry and saved as an individual FASTA file. The FASTA-formatted sequences were uploaded into Clustal Omega Multiple Sequence Alignment program (https://www.ebi.ac.uk/Tools/msa/clustalo/) and the alignment run on the sequences using the default parameters (Madeira et al., 2022). From the results, distance coordinates were downloaded using the Download Phylogenetic Tree Data feature and saved for further phylogenetic analyses. To display the phylogenetic tree, the data were imported into the interactive Tree of Life (iTOL; https://itol.embl.de/upload.cgi) (Letunic and Bork, 2021). A rectangular tree displaying the respective branch lengths was generated from the file. In addition, two subsets of sequences, including one containing *traI* genes, the most commonly named genes across the plasmid types, were chosen for analyses to determine the similarities of plasmid genes with the same name. The second subset included genes that were predicted to encode VirD4-like ATPases to determine if the predicted common gene function had tighter phylogenetic relatedness than other subsets (Gunton et al., 2005; Raleigh and Low, 2013; Christie et al., 2014; Guglielmini et al., 2014; Foley et al., 2021).

### Analysis tools development

Two different analysis tools were developed to utilize the plasmid database. The first one is used to predict and compare the putative plasmid transfer genes that are present in multiple sequenced strains and is referred to as the Plasmid Transfer Factor Comparison tool. This tool was developed using simple matching algorithms utilizing a BLAST-based strategy (Altschul et al., 1990; Altschul et al., 1997). The input files from two or more FASTA-formatted sequence files selected from the user’s workstation that are uploaded and compared to the gene sequences in the plasmid transfer factor dataset (Supplementary Table S1). The output of the comparison is a table that provides a binary matrix of the presence or absence of the transfer genes in the sequences. For a gene to be called present in the sequence, it must meet the Expect (e)-value cutoff of 10^−3^, which is the threshold used in NCBI BLAST searching (https://biopython.org/docs/1.76/api/Bio.Blast.Applications.html). To assess the function of the Plasmid Transfer Factor Comparison tool, a convenience set of 159 previously sequenced *Salmonella enterica* strains (Khajanchi et al., 2017; Han et al., 2018; Kaldhone et al., 2018; Khajanchi et al., 2019; Aljahdali et al., 2020a), that had undergone plasmid replicon typing (Carattoli et al., 2014), were analyzed to assess congruence of the results of plasmid types identified using plasmid replicon typing to the types of transfer genes that were identified using the developed database (Supplementary Table S2). This set of analysis allows for evaluation of whether factors for one plasmid type are identified as present, even though the particular plasmid type was not identified in the strain with PlasmidFinder. In addition, the sets of previously characterized IncA/C, IncB/O/K, IncFIB, IncHI1, and IncI1 plasmids (100 each) that were analyzed previously (Algarni et al., 2022a) were tested to compare the diversity of transfer genes within a particular group of plasmids and determine the congruence between the identified plasmid types and the predicted transfer genes for these groups.

A second tool termed the Plasmid Transfer Factor Profile Assessment tool provides more detailed information on the plasmid transfer genes identified within a submitted FASTA file. In this tool, a single FASTA file is uploaded and the resulting output is a detailed table containing the names of the plasmid transfer genes, the % identity to the reference sequence, number of mismatched base pairs, location of the gene in both the query and reference sequences, e-value, and bit score of the gene. The e-value cutoff for inclusion in the gene table is 10^−3^. To determine the level of sequence, overlap between the genes in the different plasmid types, a FASTA file containing all the database reference sequences was uploaded into the Profile Assessment tool and analyzed. Sequence identities were compared to determine if the genes matched to other sequences besides themselves, which would indicate non-specific targets. In addition, to evaluate and demonstrate the function of the tool with “real world data,” we utilized the WGS data from 10 *S*. *enterica* isolates that were randomly selected from the group of 159 strains above that had previous plasmid typing results (Khajanchi et al., 2017; Han et al., 2018; Kaldhone et al., 2018; Khajanchi et al., 2019; Aljahdali et al., 2020a) and were analyzed with the system to determine the predicted genes present.

## Results

### Development of a bioinformatics tools for construction and selection of plasmid transfer factor database website

**FIGURE 1:**
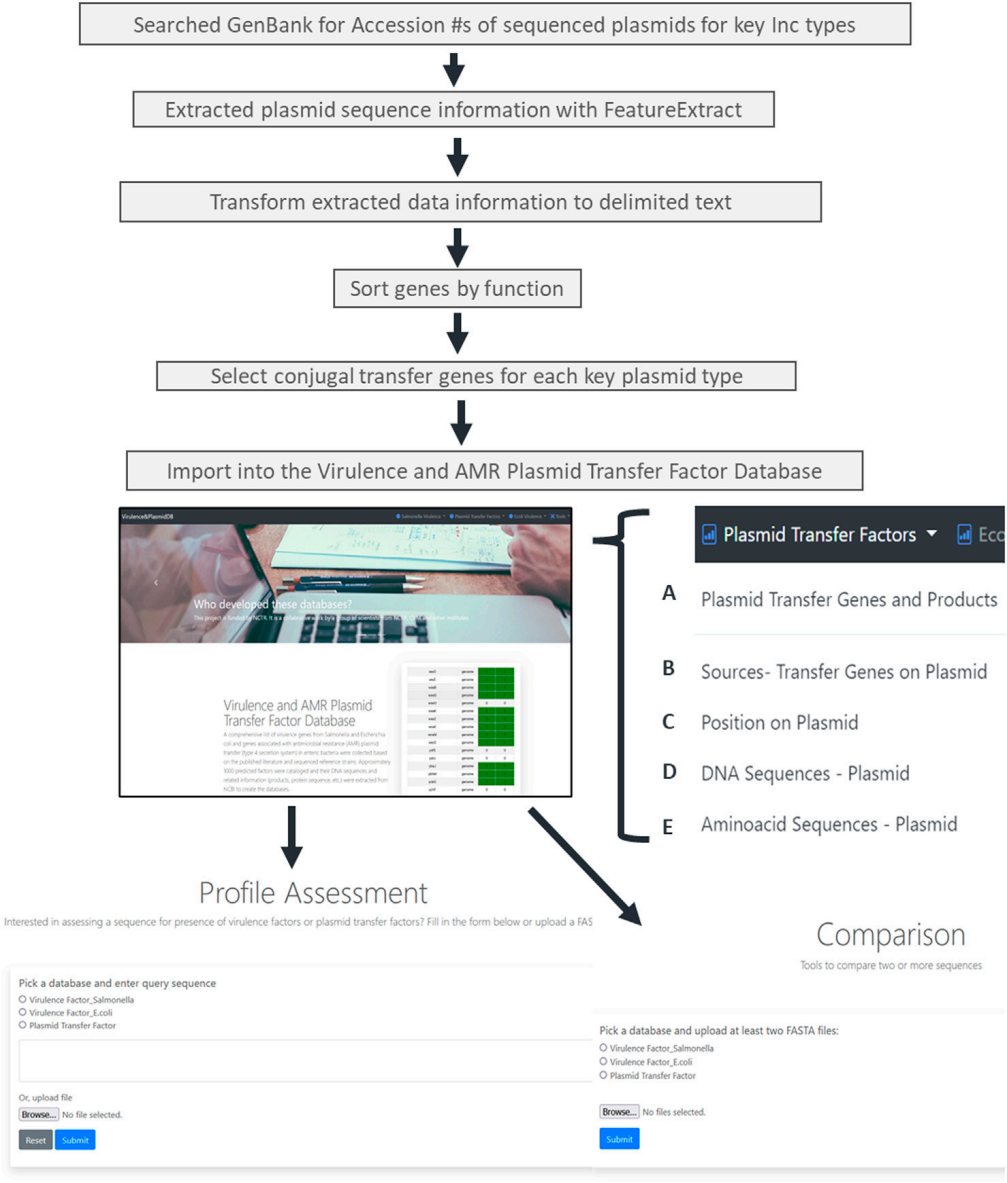
The workflow for the creation of the plasmid transfer factor database which is part of the larger virulence and amr plasmid transfer factor database, that include tools for the identification of putative virulence factors in *Salmonella* and *Escherichia coli*. The plasmid transfer factor database, shown on the bottom of the figure, is the focus of this study, contains the data on the plasmid transfer related genes (bottom right) and two analysis tools, the plasmid transfer gene profile assessment tool and plasmid transfer gene profile comparison tool (bottom left and center, respectively). The database information contains five different tabs with associated transfer factor data: **(A)** the plasmid transfer genes and products contains information for the database key for each reference genes in the database, the gene name, locus tag and DB_Xref information. The key serves as the link for each of the other tabs. **(B)** the sources-transfer genes on plasmids tab contains the key and information related to organism, strain, serovar, subspecies, culture collection, and taxon ID; **(C)** the position on plasmid link includes the Genbank accession number with a corresponding hyperlink to Genbank entry, and the DNA stand, start and end position for the gene. The final two links contain the DNA sequence (DNA sequences-Plasmid; **(D)** and predicted amino acid sequences and protein ID **(E)** of the protein encoded.

An overview of the plasmid transfer factor database workflow is shown in Figure 1. We identified representative plasmids from the Enterobacteriaceae across the 13 different plasmid types. When the assessment of the IncB/O plasmids was conducted, there was extensive overlap of the transfer gene sequences with the ones from the IncK plasmids; thus, these groups were combined so that the final database contains the transfer genes for 12 different plasmid groups. The results of the genes identified for the different plasmid types are shown in Table 1. To develop the plasmid transfer factor database, plasmid sequences from the different host species of enteric bacteria were utilized to build the set of reference genes for the analysis tools (Supplementary Table S1). This reference set contains 337 genes across the 12 different plasmid groups. A multiple sequence alignment of nucleotide sequences of the transfer genes across the different plasmid type replicons was completed using Clustal Omega and the results demonstrated considerable levels of diversity among the different sequences (Supplementary Figure S1). In general, the transfer genes of the IncHI1 and HI2 clustered most closely together, as did the IncFIA and FIB genes.

Among the gene names identified, there were significant overlaps in the names of the genes across the different plasmid types. For example, genes identified as *traI* were noted in 11 of the 12 different plasmid types (Table 1). This overlap of gene names led to some challenges in that in most cases the genes had quite divergent sequences (Supplementary Figure S2), with the exception of the aforementioned IncB/O and K plasmids that have subsequently been combined in the database. In addition, we looked at a group of sequences where the proteins are predicted to have a common function across different plasmid types. This analysis, which is shown in Supplementary Figure S3, examined genes that are predicted to encode the VirD4-like ATPases in the T4SS of Gram-negative organisms (Gunton et al., 2005; Raleigh and Low, 2013; Christie et al., 2014; Guglielmini et al., 2014; Foley et al., 2021). There was diversity among the sequences of the amino acids of the proteins with a common function, with the exceptions of the amino acid sequences of TrbC from IncB/O, K and I1 and to a lesser extent TrbC from IncM plasmids. Similarly, the TraD amino acid sequences of the IncFIA and FIB showed similarity to one another and to some extent to TraJ and TrwB from IncN and W plasmids, respectively. The VirD4 reference sequence (NCBI accession number WP_032488284.1) did not display a similar amino acid (>30%) sequence to the other predicted VirD4-like ATPases. The closest protein to the VirD4-like ATPases in the reference sequence was the TraG protein from IncP plasmids (Supplementary Figure S3).

**FIGURE 2:**
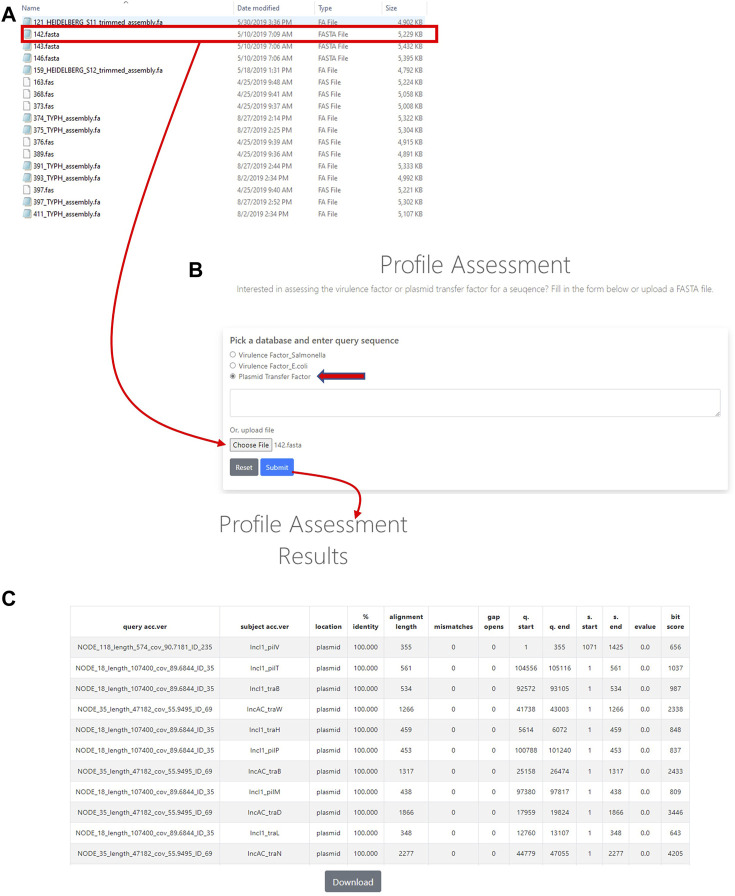
The plasmid transfer factor profile assessment (profile assessment) tool is used to assess the plasmid transfer genes in an isolate. To start the analysis, a single sequence (FASTA) file of the isolate is selected **(A)** and plasmid transfer factor database (indicated by the red arrow) is picked. Then the sequence uploaded into system **(B)** and a BLAST-based analyses is conducted. When a gene is identified information related to identity to reference, locations, etc. are provided for all the genes present **(C)**. A download button is provided at the bottom of the output to facilitate further analyses in different programs.

In order to utilize the data that was curated in the initial part of the study, tools were developed to analyze WGS data. The first of these tools was termed the Plasmid Transfer Factor Profile Assessment tool (virulence.fda.gov/tools/virulenceassess). An overview of the analyses processes is shown in Figure 2. The second tool that was developed was named Plasmid Transfer Factor Comparison tool (virulence.fda.gov/tools/virulencecompare), which is highlighted in Figure 2 and allows for the comparison of multiple different FASTA files simultaneously to give a presence/absence matrix for the comparison of the comprehensive transfer gene profiles among multiple enteric bacteria. The plasmid analyses tools described here are part of the larger Virulence and Plasmid Transfer Factor Database (virulence.fda.gov) that also contains tools for the identification of putative virulence genes in *Salmonella enterica* and *Escherichia coli.* The use of the *Salmonella* database has been reported in multiple studies (Aljahdali et al., 2020b; Tate et al., 2022).

### Assessment of the plasmid transfer factor profile assessment tool

To assess the specificity of the genes for particular plasmid types, we analyzed a file containing all of the sequences that are part of the plasmid gene database. All of the genes mapped back with 100% identity to themselves as expected, while 96 of the factors also had similarity (≥75%) with another gene in the database (Supplementary Table S3). For these 96 genes, a similarity grid was generated to compare the similarities (Figure 4). The greatest level of overlaps was between the IncFIA and IncFIB transfer genes. Additionally, ten *S. enterica* isolates from the set of 159 (Khajanchi et al., 2017; Han et al., 2018; Kaldhone et al., 2018; Khajanchi et al., 2019; Aljahdali et al., 2020a) analyzed to assess the comparison tool were used for the evaluation of the Profile Assessment tool. These isolates had a range of plasmids from none detected (MDH24) up to 5 different ones (470). As is shown in Supplementary Table S4, the transfer genes for the corresponding plasmids were detected in the strain, along with their percent identity to the reference sequences (Supplementary Table S4), alignment length, mismatches and gaps, and alignment information. For the plasmid types in our transfer database, when the corresponding plasmids were detected by PlasmidFinder, their representative transfer genes were detected with the Profile Assessment Tool. In some cases, there were transfer genes detected for plasmid types that were not detected with PlasmidFinder, these included the detection of multiple IncFIA-associated genes that were overlapping with the IncFIB genes, isolate N032 which had multiple IncI1-associated genes, and several of the IncA/C-associated genes (particularly *traJKMPQRSTY*) in multiple strains (426, CVM16, N030, and N032). Coincidently, the strains in this later group are all positive for IncFIC(FII) plasmids, which may be the source of the overlapping IncA/C transfer genes. There were also some other overlapping genes that appear to be “false positive” calls, since their identity values were relatively low (<80%) and their alignments overlapped with corresponding genes detected in plasmids identified by replicon typing (Supplementary Table S4).

### Assessment of the plasmid transfer factor comparison tool

The Plasmid Transfer Factor Comparison tool is highlighted in Figure 2 and facilitates the comparison of multiple different FASTA files simultaneously to provide a presence/absence matrix for the comparison of the transfer gene profiles among multiple strains. To assess this tool, two sets of data including the cohort of IncA/C, IncB/O/K, IncFIB, IncHI1, and IncI1 plasmids previously described (Algarni et al., 2022a), as well as WGS data from the 159 *Salmonella* strains that were previously sequenced, were analyzed.

Figure 5 shows the overview of results of the analyses of the IncA/C, IncB/O/K, IncFIB, IncHI1, and IncI1 plasmid sequences. In all cases, at least one of IncA/C transfer genes was detected and, 84 (84%) carried the IncA/C *traABCDEFGHILNUVW* and *trhF* genes. Conversely, *traKMPQRST* were detected in only one of the plasmids, while *traJ* and *traY* were not detected in any of the 100 sequences. In the second most common genotype (N = 8, 8%), the plasmids carried the *traABDEFGHILV* genes and the third most common genotype (N = 2, 2%) carried the *traABCEFGHILV* genes (Figure 5).

When we initially analyzed the IncB/O plasmids, with at least one IncB/O gene being detected in all 100 plasmids analyzed. A key observation was the presence of a mirror image of the gene profiles for the IncK genes with those of the IncB/O genes, thus we combined them into the IncB/O/K group which is consistent with the recent replicon typing schema that identifies the plasmid type as IncB/O/K/Z. There was a diversity of different genotypes, with the most common (N = 46, 46%) being the presence of all of the IncB/O/K transfer genes (Figure 5). Another common set of genotypes was the absence of multiple genes in the *pilK-U* transfer gene region. The results of the IncHI1 and IncI1 plasmid analyses were similar to those of the IncA/C and IncB/O/K, with all of the plasmids having at least one of the associated transfer genes (Figure 5). For the IncHI1 plasmids, the vast majority (N = 96, 96%) contain all of the transfer genes and for the IncI1 plasmid the vast majority have either all of the genes (N = 39, 39%) or missing *traD, pilI* and *pilJ* (N = 48, 48%) (Figure 5). The results of the IncFIB plasmids were more variable, with 86 (86%) having at least one of the IncFIB-associated transfer genes (Figure 5). The gene profiles were also quite varied, with 33 different genotypes and the largest grouping with 10 (11.6%) of those isolates with at least one transfer gene.

The second set of analyses explored a diverse group of WGS data from *S. enterica* isolates previously characterized and representing multiple serotypes and plasmid replicon types (Khajanchi et al., 2017; Han et al., 2018; Kaldhone et al., 2018; Khajanchi et al., 2019; Aljahdali et al., 2020a). In most cases, the transfer genes identified correlate well with the plasmid replicon typing with some noted exceptions (Figure 6; Supplementary Table S2). In all cases where IncA/C, HI1, HI2, I1, and I2 plasmids were identified by replicon typing, at least one corresponding transfer associated genes was present. There were similar results for the IncFIA and IncFIB positive strains, with all but one FIA and two FIB strains containing corresponding transfer genes. There were several cases where IncFIA transfer genes were detected, but the strains did not have the corresponding replicon, which is likely due to the similarity of sequence with several genes present on the IncFIB plasmid replicon (Figure 4).

## Discussion

The database and tools generated in this study will allow researchers to further utilize the data generated from WGS studies and gain a fuller understanding of genetics and diversity of plasmid transfer systems in enteric bacteria. The overall project was conducted to develop and evaluate a plasmid transfer gene database and associated tools known as the Plasmid Transfer Factor Profile Assessment and Plasmid Transfer Factor Comparison tools. These reported tools and database represent portions of the larger FDA Virulence and Plasmid Transfer Factor Database that also includes analytical tools of *Salmonella* and *E. coli* putative virulence genes. These virulence database efforts are not directly evaluated in the current manuscript, but can provide complementary data when the virulence genes are carried on plasmids. The plasmid database was created by examining whole plasmid sequences that were deposited in GenBank for some of the key plasmid replicon types that are associated with antimicrobial resistance in *Salmonella* and other enteric bacteria (Carattoli, 2009; Rozwandowicz et al., 2018; Algarni et al., 2022a). The plasmid data were extracted from over 1,200 different plasmids, the genes associated with the conjugal transfer of the different types of plasmids were identified, and the DNA and amino acid sequences were downloaded and used to create the Plasmid Transfer Factor Database (Figure 1).

**FIGURE 3:**
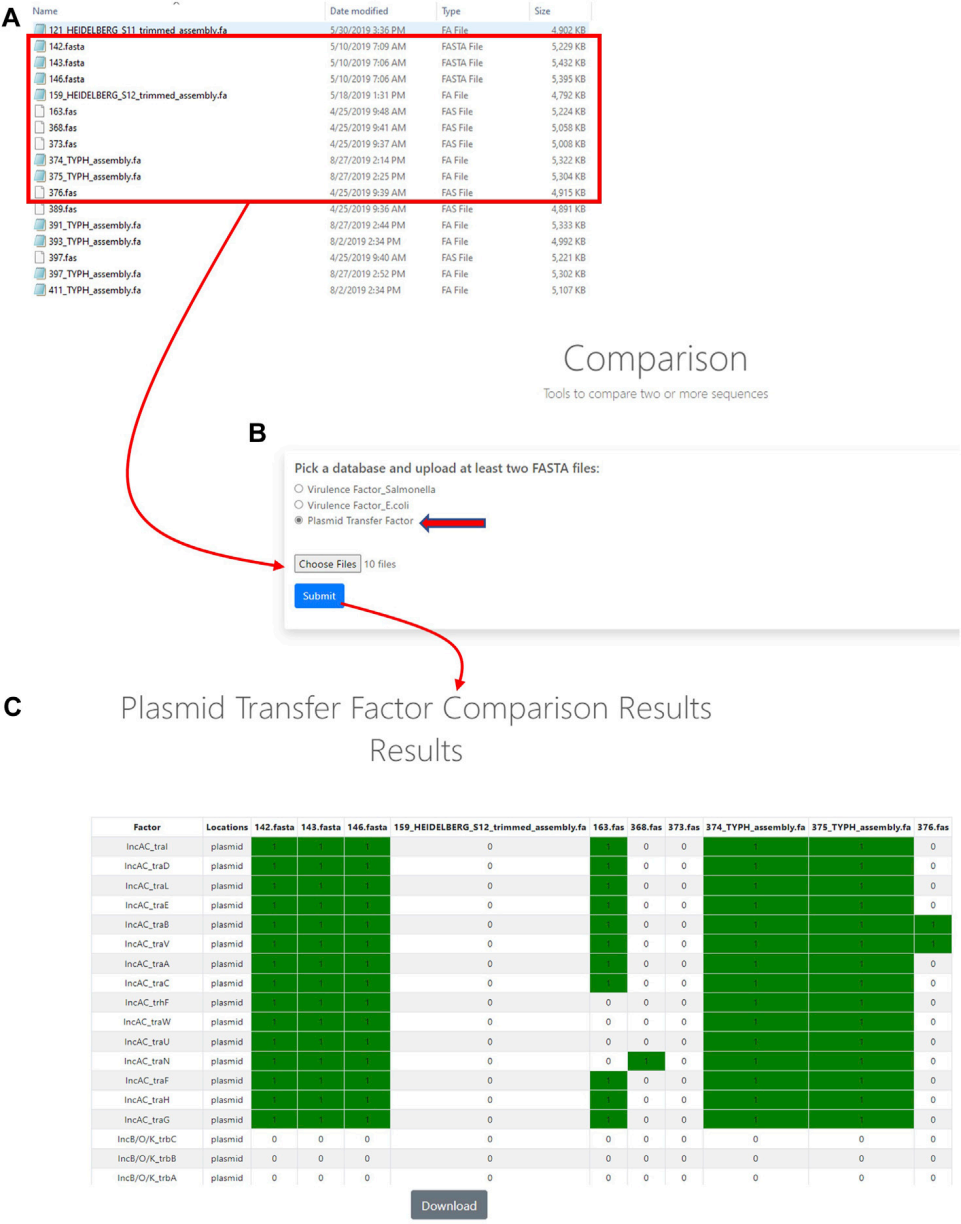
The plasmid transfer factor comparison tool is used for the comparison of multiple different FASTA files simultaneously to give a presence/absence matrix for the comparison of the comprehensive transfer gene profiles among multiple enteric bacteria. To start the analyses, multiple sequence (FASTA) files of interest are selected for uploading **(A)** and plasmid transfer factor database (indicated by the red arrow) is selected to upload the sequences into system **(B)** and a BLAST-based analyses is conducted to identify the predicted genes present. When genes are detected a resulting presence/absence matrix is generated to facilitate comparison among strains **(C)**. These analyses can be downloaded as a tab-delineated file using the download button and extracted for further phylogenetic analyses.

After initial rounds of assessment, the final set of transfer associated genes included 337 sequences from 12 different plasmid types (Table 2, Supplementary Table S1). A key feature of the database is that as the study of antimicrobial resistance plasmids in enteric bacteria continues, this initial set of transfer genes can be expanded to include additional plasmid types and transfer genes in order to meet the needs of the scientific community. Figures 2, 3 show the basic interfaces and outputs for the Plasmid Transfer Factor Profile Assessment and Plasmid Transfer Factor Profile Comparison tools, respectively. The output of the Plasmid Transfer Factor Profile Assessment tool can be exported into an Excel spreadsheet format for easy sorting and data analyses. The ability to show the % identity to reference and the start/stop locations for the genes is quite valuable to understand the order of the genes within the transfer operon and whether the genes are conserved or not.

**FIGURE 4:**
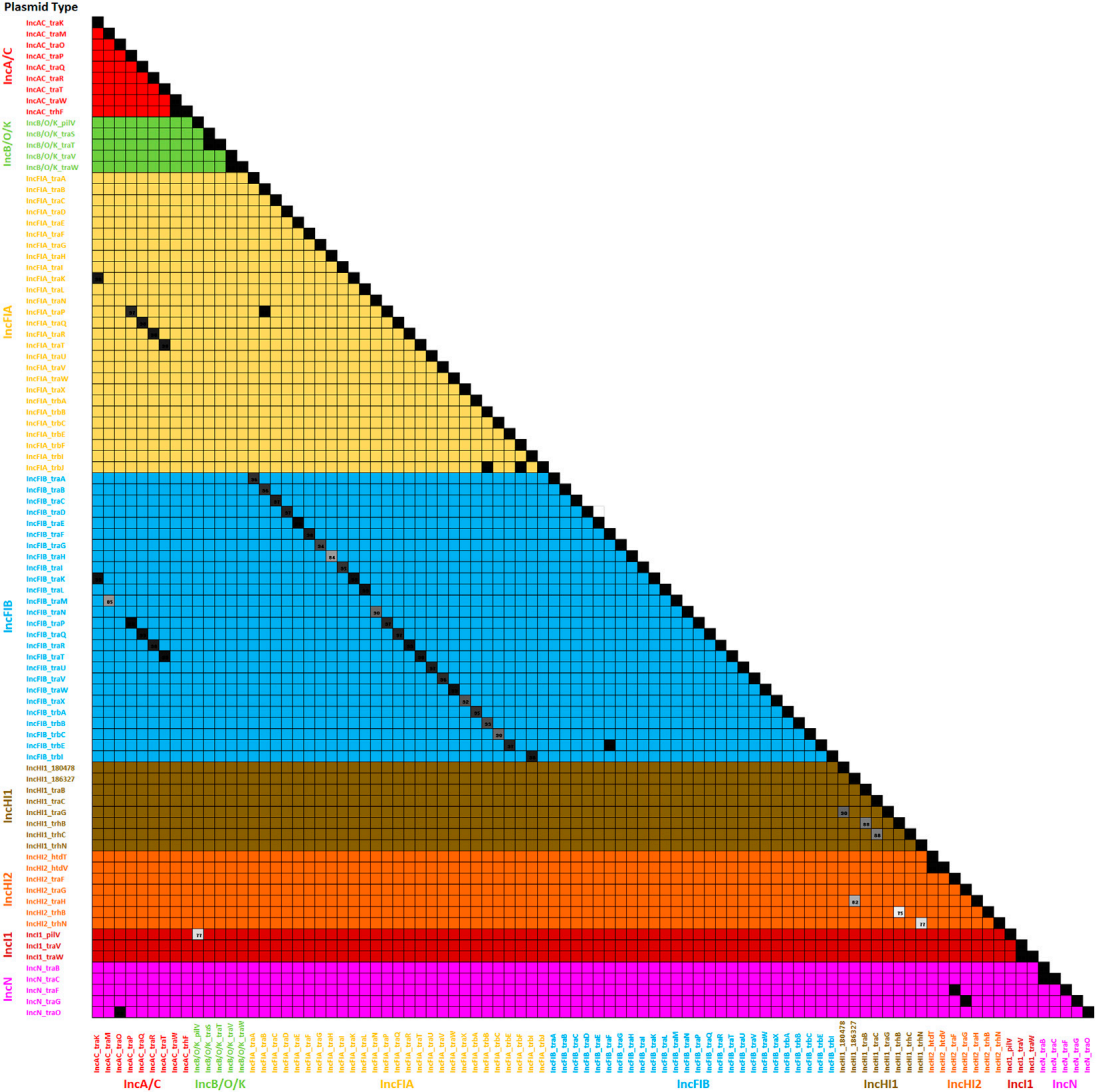
The plasmid transfer factor assessment tool analyses of the genes in the database. This figure is a similarity matrix of the 90 of the factors that had similarity with another gene in the database (see Supplementary Table S3). When there was overlap in the sequence identity between two genes, the similarity was indicated with a number in the box. The color of the numbered boxes ranges from 84% identity (light grey box) to the black boxes with 100% identity.

**FIGURE 5:**
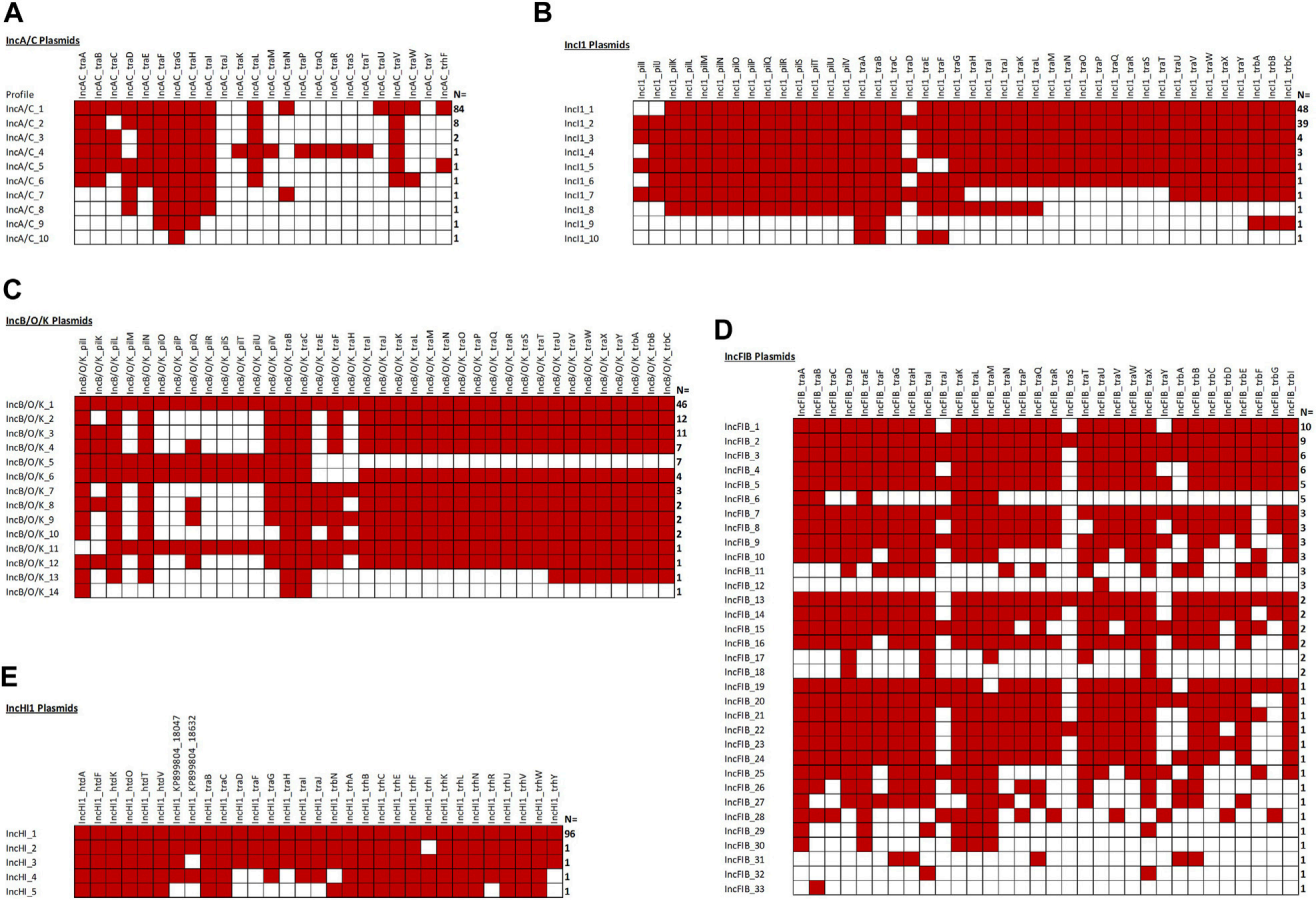
The plasmid transfer factor comparison tool for the IncA/C **(A)**, IncI1 **(B)**, IncB/O/K **(C)**, IncFIB **(D)**, and IncHI1 **(E)** plasmids. One hundred of each plasmid type were analyze and the number at the right indicates the number with the particular genotype. A red box is positive for the gene. Note, 14 IncFIB plasmids lacked any of the associated genes.

**FIGURE 6:**
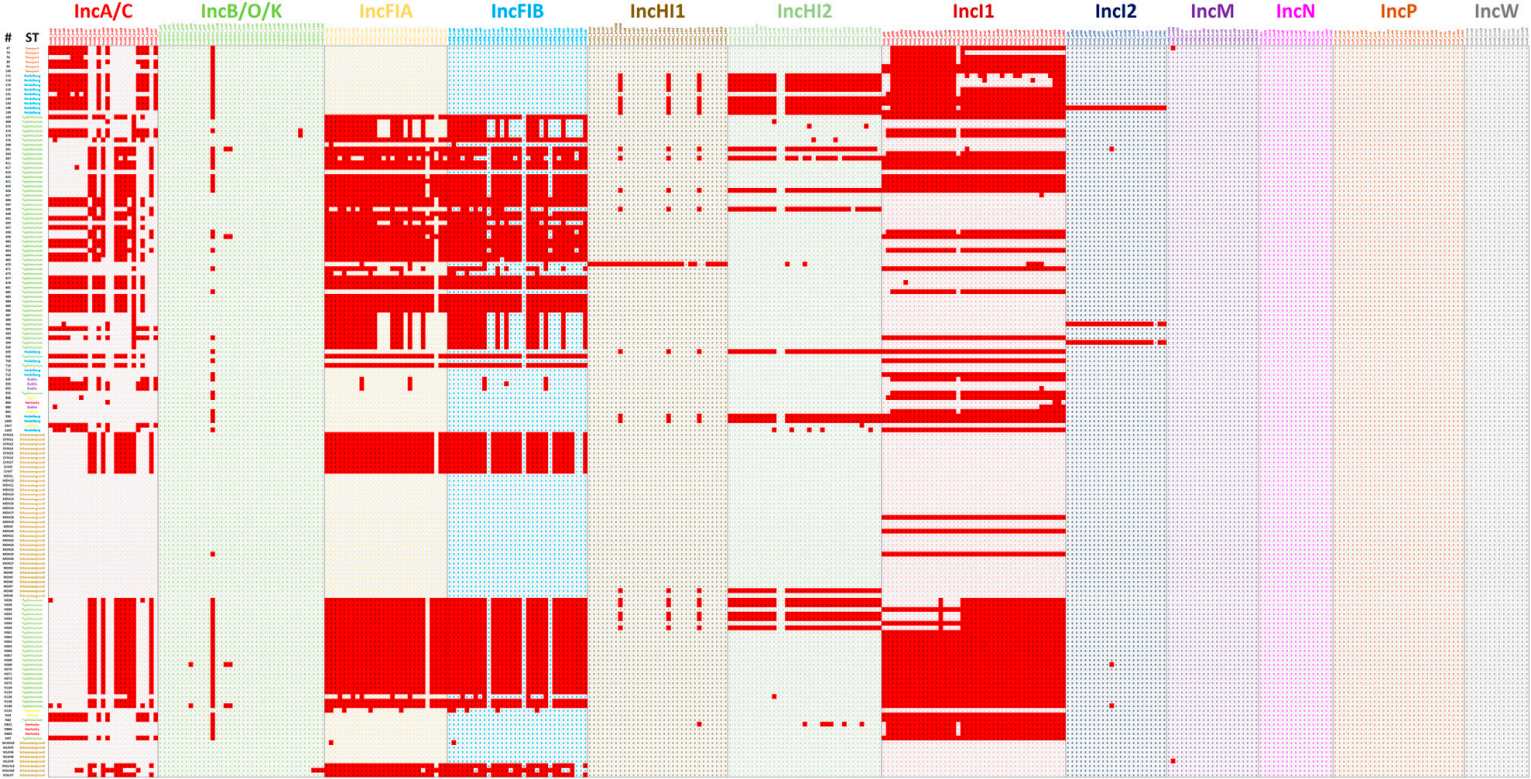
The plasmid transfer factor comparison tool for 159 previously sequenced strains to assess the overall function of the database to predict the presence of transfer genes. The ID column contains the strain numbers and the ST column the different serotypes, with the different serotypes differentiated by different color blocks. The detailed information on the results presented are shown in Supplementary Table S2.

**FIGURE 7:**
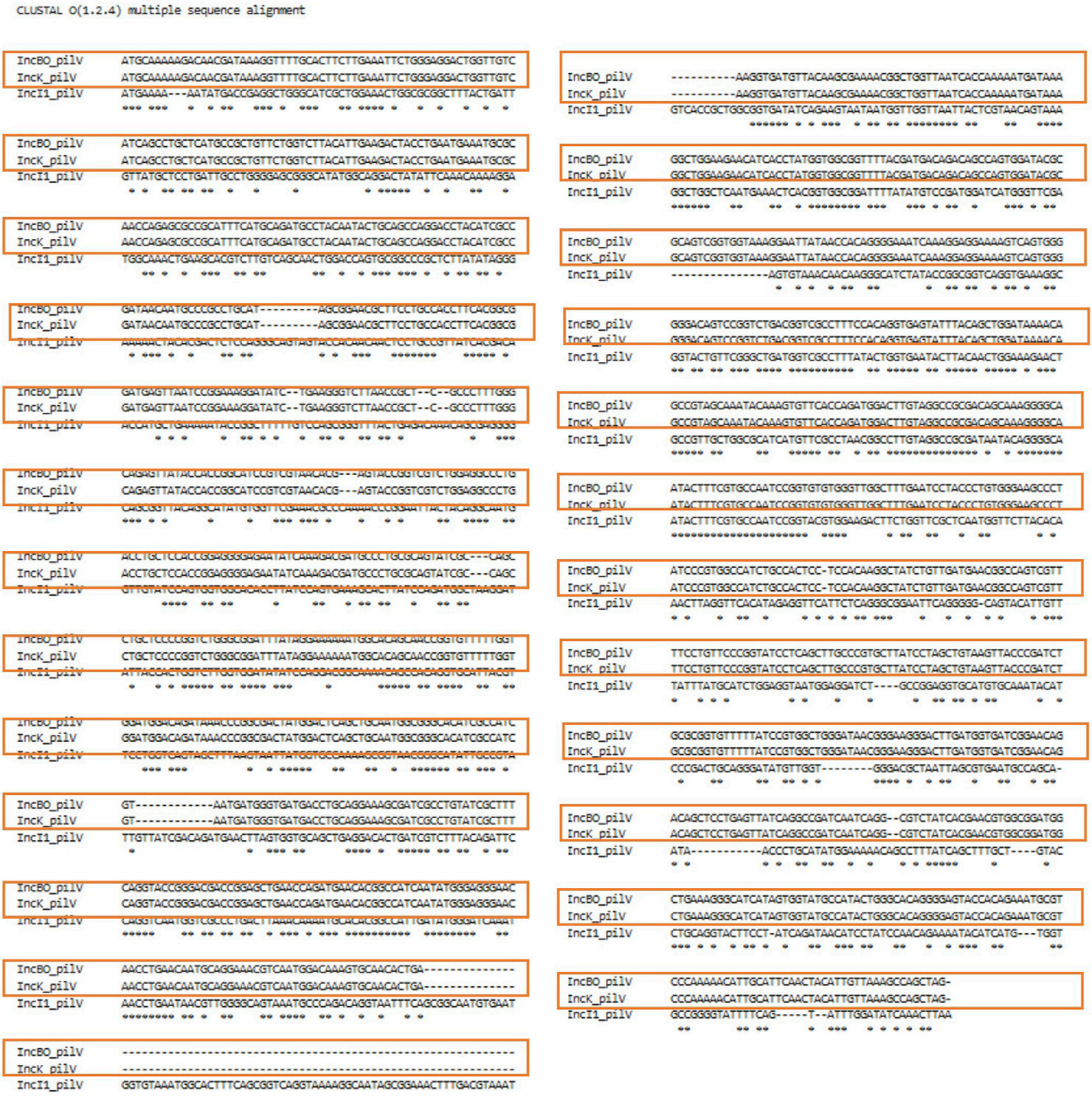
Clustal Omega alignment for the sequences of the *pilV* genes of the IncB/O, K and I1 plasmids. The orange boxes highlight the 100% overlap of the nucleotides between the IncB/O and IncK *pilV* genes.

The Plasmid Transfer Factor Profile Comparison tool allows for the upload and comparison of multiple sequences simultaneously. The result is a binary output representing the presence or absence of genes in the database and can be downloaded for further analyses. An initial evaluation carried out in this study was the screening of the 100 IncA/C plasmid sequences described in our previous study (Algarni et al., 2022a) to determine specificity of the database for transfer genes within the single plasmid type. IncA/C plasmids are key elements for the simultaneous dissemination of multiple antimicrobial resistance genes (Hegyi et al., 2017; Hoffmann et al., 2017). All of the plasmids analyzed had at least one IncA/C associated transfer gene, although not all genes were present in each of the plasmids (Figure 7). This diversity of IncA/C transfer genes has been described previously and may play a role in the ability of IncA/C plasmids to transfer among strains (Han et al., 2018). The IncHI1 plasmids displayed much less diversity of transfer genes, with 96% of the plasmid carrying all of the IncHI1 genes incorporated in the database (Figure 5). The IncI1 plasmids were dominated by two major genotypes, one containing all the affiliated genes (39%) and the other lacking the *pilI, pilJ* and *traD* genes. When the IncFIB positive strains were examined 86 (of 100) isolates had at least one of the IncFIB-associated transfer genes. There was extensive overlap with the IncFIA genes, which was not too surprising since there was extensive sequence overlap between several of the genes (Figure 4). The assessment of the Comparison tool was broadened to evaluate 159 *Salmonella enterica* strains that had undergone WGS and analyzed using the PlasmidFinder tool (Khajanchi et al., 2017; Han et al., 2018; Kaldhone et al., 2018; Khajanchi et al., 2019; Aljahdali et al., 2020a). In general, when a plasmid type was identified by PlasmidFinder, at least some of the corresponding transfer associated genes were detected (Figure 6; Supplementary Table S2). A couple of exceptions were with the IncF plasmids, where in several instances both IncFIA and FIB plasmid replicons were detected, but only contained the IncFIB transfer genes. In several *Salmonella-*associated plasmids there appears to be a co-integration of the IncFIA replicon sequence within the backbone of the IncFIB plasmids that explains these observations (Hsu et al., 2019; Liu et al., 2021). Another interesting observation was that the strains carrying the IncFIC(FII) plasmids often had multiple “IncA/C” transfer-associated genes *traJKMPQRSTY* (Supplementary Figure S2) that were rarely, if ever, detected in the 100 IncA/C plasmids screened in Figure 5. The determination that these genes are included among the IncA/C genes may need to be re-evaluated and may merit the addition of the IncFIC plasmid genes.

In order to evaluate whether the different genes were appropriate for incorporation in the Plasmid Transfer Factor Database, a series of different analyses were conducted. Initially a global phylogenetic analysis of all the transfer associated genes selected for the database was conducted in order to see if there was significant overlap in the genes from the different plasmid types. As was shown in Supplementary Figure S1, there was very little overlap in the genes from the different plasmid types. A key exception to this diversity was observed for the IncB/O and IncK plasmid groups when they were initially evaluated as separate plasmid groups. In these analyses, each set of genes for IncB/O and K were identical. To confirm that combining the IncB/O and K genes could be combined, we completed an alignment with the IncB/O and K genes using Clustal Omega which showed that there was extensive overlap of the gene sequences, with the IncB/O and K sequences being identical, this observation is highlighted with the red boxes in the alignments of their *pilV* genes (Figure 6). Likewise, the significant overlap of the IncBO and K plasmid types was quite evident when we screened nearly 100 identified IncB/O plasmids with our original database iteration. In each of these cases the genes identified in the IncBO plasmids were mirrored by those of the same name in the IncK plasmids. Additionally, with the *pilV* gene, there was also some cross positivity with the IncI1 and to a lesser extend IncI2 plasmids. This cross reactivity was also observed when the WGS data from the 10 isolates that were analyzed with the Assessment Tool where the IncI1 positive strains had similarity to the *pilV* genes from the IncB/O/K plasmids (Supplementary Table S3). To confirm the similarity of the IncB/O, K and I1 plasmids further, we looked at the *pilV* gene sequences in each of these types and carried out a sequence alignment (Figure 7) and observed that the genes that were identical for the IncB/O and K plasmids and the I1 plasmids had significant overlap. The *pilV* gene encodes for an adhesin that is present at the tip of the conjugal pilus (T4SS) that interacts with the lipopolysaccharide (LPS) on the recipient cell during conjugation in liquid environments (Ishiwa and Komano, 2000; 2003; Foley et al., 2021). This overlap between the transfer genes was noted elsewhere in research exploring IncI-related plasmids. Zhang et al. (2019) undertook comparative genomics analyses of multiple IncI, B/O, K and Z plasmids and found high degrees of similarities (>95%) across the transfer genes of IncBO and K plasmids (Zhang et al., 2019). Likewise, PlasmidFinder now groups IncB/O, K and Z as a single replicon type, IncB/O/K/Z, in their analyses (Shirakawa et al., 2020; Darphorn et al., 2021). Thus, the current version of the database was merged into a single plasmid type in the Plasmid Transfer Gene Database (IncB/O/K) that is more consistent with the current plasmid typing tools, such as PlasmidFinder.

Another feature of the global phylogenetic analysis was the identification that genes with identical names are not always closely related in the phylogenetic trees. For example, the *tral* gene was the most common gene present across plasmid replicon types, detected in all plasmid types with the exception of IncW. When the phylogenetic analyses were conducted on just the *tral* genes from the different plasmid types, there was significant diversity in the gene sequences (Supplementary Figure S2). This is likely due to the naming issues with the genes in different plasmid types being named in an *ad hoc* fashion as the secretion systems were characterized initially. For example, in the IncF plasmids, TraI serves as a relaxase and there is a domain responsible for conjugative repression that resides in the central region of TraI, which plays a role in the regulation of conjugation (Haft et al., 2006). Moreover, TraI in the IncF-like plasmids were observed to serve as translocation signals mediating type 4 secretion (Zatyka and Thomas, 1998; Waksman, 2019). In contrast, in the IncI-like plasmids, TraI is part of a multiprotein structural complex in the outer membrane that helps to stabilize the conjugal pilus (Foley et al., 2021). As these two examples show, the naming of the plasmid transfer genes is quite confusing and should probably be reassessed to help clarify the nomenclature. One possible way to rectify these issues could be to use naming approaches such as shown with the IncW plasmids, where the genes are named with the precursor “*trw*”, which indicates that there are transfer genes (*tr*) from IncW (*w*) plasmids. It appears that there is some suggestions in this direction as some of the IncHI1 and IncHI2 transfer genes are also annotated as *“trh_*”. A possible challenge with this approach is the long-standing existing nomenclature that is embedded in the literature that would need to be cross-referenced with a revised nomenclature.

As the *traI* example showed, the naming of plasmid factors is quite variable. When we looked at the proteins that are predicted to have the same function, they are often named quite differently. For example, when we look at the predicted homologs of VirD4 ATPases or type four coupling protein (T4CP) from prototypical T4SS of the bacterium *Agrobacterium tumefaciens* (Zatyka and Thomas, 1998; Raleigh and Low, 2013; Christie et al., 2014; Foley et al., 2021), there are several different gene/protein names (Supplementary Figure S3). As is shown in Supplementary Figure S3, the level of amino acid similarity between the reference VirD4 and the other predicted ATPases was quite divergent. Some of the predicted ATPases displayed similarity including TrbC belonging to IncB/O and K, IncI1 and IncM and TraD from IncFIA and FIB, which also displayed similarity to TrwB and TraJ belong to IncW and IncN, respectively. However, these protein sequences that are clustered together are divergent from the other predicted ATPase genes from the other plasmid types. In general, T4CPs of Gram-negative conjugation machines stimulate the ATPase activity of the coupling protein, which is required to initiate the relaxosome complex of the T4SS (Hegyi et al., 2017). The T4CPs influences the nucleotide binding properties that facilitate loading of plasmid DNA into the T4SS for transfer (Alvarez-Martinez and Christie, 2009; Raleigh and Low, 2013).

The Plasmid Transfer Factor Profile Assessment Tool proved to be quite useful in understanding the genetic relatedness of the different transfer genes. For example, the analyses shown in Supplementary Table S3; Figure 4 highlights the similarity of several of the transfer genes among the different plasmid types. For example, the IncA/C genes that overlap with the IncFIA and FIB plasmids are those that were commonly detected among the IncFIC(FII) plasmids, but not common among the IncA/C positive strains, indicating that they may be misclassified as IncA/C-associated genes. Another key feature of the Profile Assessment Tool is that it provided the locations of the genes within the sequencing contig (or whole plasmid, if closed) that can be integrated with location data from other databases such as AMRFinderPlus, IntegronFinder, and the complementary virulence gene datasets that are part of the FDA Virulence and Plasmid Transfer Factor Database to understand what genes are located on what plasmids (Feldgarden et al., 2019; Algarni et al., 2022a; Neron et al., 2022). These data can aid in a better understanding of the horizontal gene transfer potential for antimicrobial resistance and/or virulence determinants on specific plasmids. The presence of the complement of plasmid transfer genes may indicate the potential for the conjugal spread of the plasmids under different scenarios. In the near future, with more plasmid sequencing data being analyzed using our database, we may be able to identify the key genes or transfer factors that play important roles in facilitating plasmid transfer between various strains or bacterial species.

## Conclusion

Multidrug resistance in *Salmonella* and other enteric pathogens remains a worldwide challenge, in large part due to the dissemination of AMR by plasmids. A key challenge to mitigate AMR is trying to predict the potential for plasmid transfer and to prevent AMR spread. The Plasmid Transfer Factor Database and tools evaluated in the current project provide very extensive bioinformatics tools that enable the identification and characterization of the plasmid transfer associated genes. The database and tools allow for the detection of differences in the transfer gene content that potentially have an impact of the abilities of different plasmids to transfer, as well as identifying highly transmissible MDR plasmids, which will allow for risk assessment and develop a strategy to prevent and combat highly mobilized MDR plasmids from further spreading. Additionally, the tools allow for a direct phylogenetic comparison of replicon types and transfer genes among bacterial strains, which can help understand the origins, evaluation, and distribution of plasmids across bacterial populations.

## Data Availability

The datasets presented in this study can be found in online repositories. The names of the repository/repositories and accession number(s) can be found in the article/Supplementary Material.
