# Supplementary figures and images for: Development of an antimicrobial resistance plasmid transfer gene database for enteric bacteria
Source: Front Bioinform. 2023 Nov 14;3:1279359. doi: 10.3389/fbinf.2023.1279359 (PMC10682676; doi:10.3389/fbinf.2023.1279359)

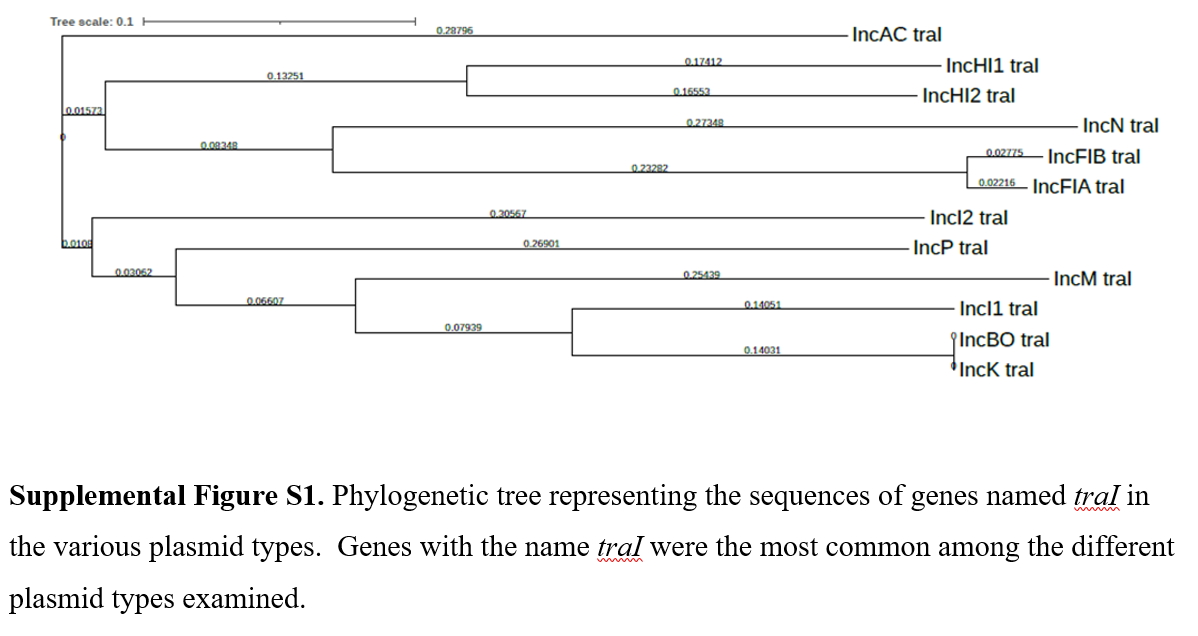

Supplement: Supplementary file 1 [file Image1.JPEG]

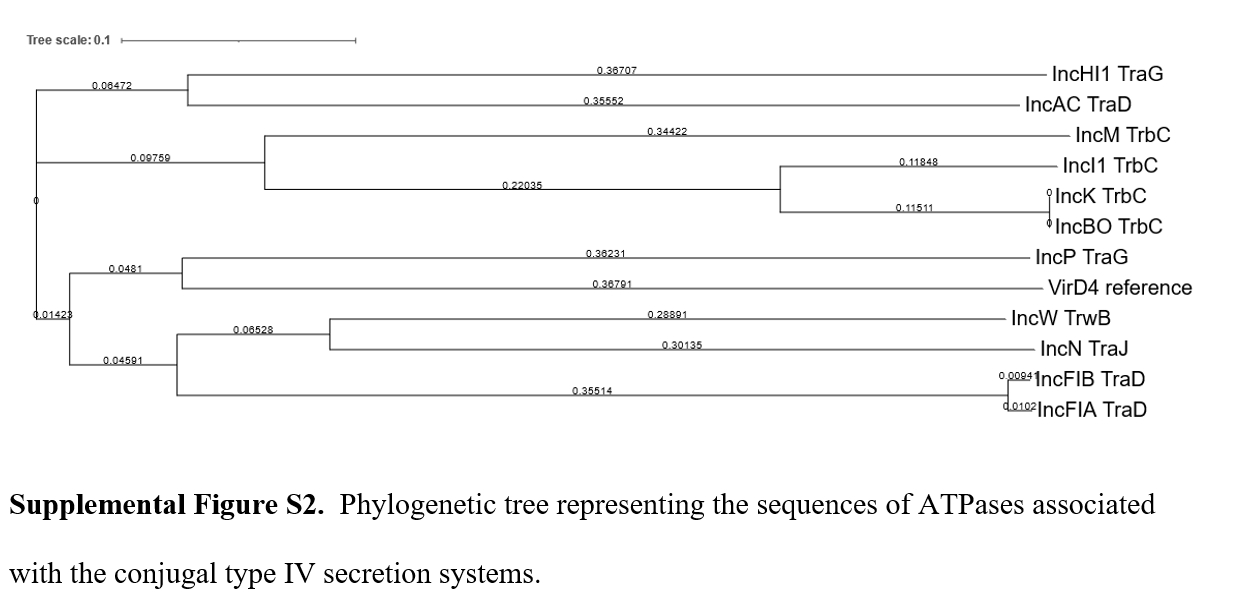

Supplement: Supplementary file 2 [file Image2.JPEG]

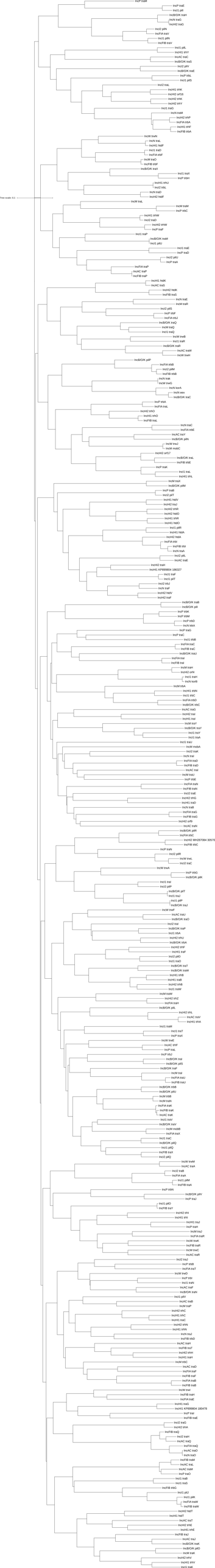

Supplement: Supplementary file 3 [file DataSheet1.PDF]
